# *Rho *iso-alpha acids from hops inhibit the GSK-3/NF-κB pathway and reduce inflammatory markers associated with bone and cartilage degradation

**DOI:** 10.1186/1476-9255-6-26

**Published:** 2009-08-27

**Authors:** Veera Reddy Konda, Anuradha Desai, Gary Darland, Jeffrey S Bland, Matthew L Tripp

**Affiliations:** 1 MetaProteomics Nutrigenomics Research Center (a subsidiary of Metagenics, Inc), 9770 44^th ^Avenue N.W., Gig Harbor, WA, 98332, USA

## Abstract

**Background:**

*Rho *iso-alpha acids (RIAA) from hops have been shown to have anti-inflammatory properties. To understand the mechanisms, we evaluated the effect of RIAA in cell signaling pathways and inflammatory markers using various in vitro models. We also investigated their therapeutic effect in mice with collagen-induced arthritis.

**Methods:**

The LPS-stimulated RAW 264.7 macrophages were used to evaluate the effect of RIAA on the NF-κB and MAPK signaling pathways; phosphorylation of ERK1/2, p38 and JNK was assessed by western blotting and NF-κB binding by electrophoretic mobility shift assays. Effect on the NF-κB activity was evaluated by the luciferase reporter assays in LPS-stimulated RAW 264.7 cells. GSK-3α/β kinase activity was measured in cell-free assays. The inhibitory effect of RIAA on inflammatory markers was assessed by measuring nitric oxide in LPS-stimulated RAW 264.7 cells, RANKL-mediated TRAP activity in transformed osteoclasts, and TNF-α/IL-1β-mediated MMP-13 expression in SW1353 cells. Mice with collagen-induced arthritis were fed with RIAA for 2 weeks. Symptoms of joint swelling, arthritic index and joint damage were assessed.

**Results:**

RIAA selectively inhibited the NF-κB pathway while having no effect on ERK1/2, p38 and JNK phosphorylation in LPS-stimulated RAW 264.7 cells. RIAA also inhibited GSK-3α/β kinase activity and GSK-3β dependent phosphorylation of β-catenin in RAW 264.7 cells. In addition, RIAA inhibited NF-κB-mediated inflammatory markers in various cell models, including nitric oxide in LPS-stimulated RAW 264.7 cells, RANKL-mediated TRAP activity in transformed osteoclasts, and TNF-α/IL-1β-mediated MMP-13 expression in SW1353 human chondrosarcoma cells. Finally, in a mouse model of collagen-induced arthritis, RIAA ameliorated joint damage as evidenced by significant reduction of the arthritis index and histology score; at 250 mg/kg-body weight, RIAA had efficacy similar to that of 20 mg/kg-body weight of celecoxib.

**Conclusion:**

RIAA may have potential as an anti-inflammatory therapeutic.

## Background

The inflammatory markers such as prostaglandin (PG) E_2_, nitric oxide (NO), tumor necrosis factor-α (TNF-α), and interleukins (ILs) play important role in chronic inflammatory diseases. Inflammation is mediated by several transcriptional factors, including NF-κB, CREB, C/EBPβ and AP-1, through the activation of multiple signaling pathways; for example, NF-κB, MAPK ERK1/2, p38 and PI3K pathways (reviewed in [1]).

In the presence of a stimulus, such as lypopolysaccharide (LPS), the innate immune response is triggered via activation of the NF-κB pathway: activation of IκB kinase (IKK) complex leads to phosphorylation of IκB and causes the degradation of the complex, which permits the dissociation and nuclear translocation of NF-κB p50/p65. NF-κB in the nucleus binds to DNA and activates inflammatory genes and proteins. Alternatively, independent of IKK activation, phosphorylation of NF-κB p65 at serine 468 by glycogen synthase kinase (GSK)-3β also activates the NF-κB pathway, and the inhibition of GSK-3β has been shown to ameliorate inflammation [2,3]. In addition, gene knockout mice of NF-κB p65 or GSK-3β showed similar phenotype and embryonic lethality caused by liver degeneration [4,5], suggesting that they share a common pathway. Hence, the current development of compounds/drugs to treat inflammatory diseases (e.g. rheumatoid arthritis, or RA) has been targeting the GSK-3/NF-κB pathway.

Rho iso-alpha acids (RIAA) are a modified extract from hops (*Humulus lupulus*) that has self-affirmed GRAS (generally regarded as safe) status as determined by an expert panel and used as flavoring/bittering agents in the brewing industry throughout the globe. Our past research suggested that RIAA had anti-inflammatory potential; RIAA dose-dependently inhibited PGE_2 _production in LPS-stimulated RAW 264.7 macrophages and reduced knee arthritic pain in humans with no reported serious adverse effects [6,7]. In addition, in contrast to nonsteroidal anti-inflammatory drugs (NSAIDs), RIAA inhibited inducible but not constitutive cyclooxygenase (COX)-2 in vitro; and in human studies, RIAA showed no effect on fecal calprotectin and urinary PGI_2_, markers used to assess gastrointestinal and cardiovascular complications [6]. Furthermore, animal oral toxicology data reveal that an RIAA-containing product (45% RIAA of 250 mg/kg/day) for 21 days showed no adverse effects in mice [8]. These results indicate that RIAA have safer, therapeutic potential to address inflammation.

To understand the anti-inflammatory mechanisms, we evaluated the effects of RIAA in cell signaling pathways and inflammatory markers using various in vitro models. We also investigated the therapeutic effects of RIAA in mice with collagen-induced arthritis (CIA).

## Materials and methods

### Materials

RIAA was supplied by Hopsteiner (New York, NY); the chemical composition of RIAA was described in [6]. Phospho-ERK1/2, phospho-p38, phospho-JNK, phospho-β-catenin anti-bodies were purchased from Cell Signaling Technology (Danvers, MA). SB216763 was purchased from Biomol (Plymouth Meeting, PA). LPS (from E. coli), anti-actin antibody, parthenolide and other analytical grade chemicals were purchased from Sigma (St. Louis, MO). Electrophoresis gels and reagents were purchased from Bio-Rad (Hercules, CA).

### Cell culture

RAW 264.7 macrophages were purchased from ATCC (Manassas, VA) and maintained in Dulbecco's Modified Eagle's Medium (DMEM) in the presence of 10% fetal bovine serum (FBS), 100 U penicillin/ml and 100 μg streptomycin/ml, according to manufacturer instructions. All test compounds were dissolved in DMSO, then diluted in serum-free media and used at a final concentration of 0.1% DMSO.

### Electrophoretic mobility shift assays (EMSA)

RAW 264.7 cells were sub-cultured and grown overnight in 6-well plates at a density of 2 × 10^6 ^cells per well, and incubated in the absence or presence of RIAA for 1 h followed by LPS (1 μg/ml) stimulation for 2 h. Nuclear extract was prepared as previously described [9] with modifications. Briefly, cells were lysed with lysis buffer containing 10 mM Hepes-KOH (pH 7.9), 0.1% NP-40, 10 mM KCl, 1.5 mM MgCl_2_, and protease inhibitor cocktail (Amersham Biosciences, Piscataway, NJ) for 15 min on ice and centrifuged at 10,000× for 10 min. Cell pellet was washed with the lysis buffer, resuspended in nuclear extract buffer containing 20 mM Hepes-KOH, 25% glycerol (v/v), 1.5 mM MgCl_2_, 420 mM NaCl, 0.2 mM EDTA and protease inhibitor cocktail, and sonicated (2 × 10 sec at 60% power level). The samples were centrifuged at 10,000× for 10 min and the nuclear extract was stored at -80°C until analysis. For DNA binding activity, 5 μg of the nuclear extract was incubated with ~3 × 10^4 ^cpm of [^32^P]ATP -labeled NF-κB consensus oligonucleotide (5'-AGTTGAGGGGACTTTCCCAGGGC) at room temperature for 20 min. This EMSA probe has been previously shown to be specific for NF-κB [10]. Following electrophoresis at on 5% nondenaturing acrylamide gel, the gel was dried and exposed to X-ray film and developed by autoradiography.

### Western blot analysis

RAW 264.7 cells were grown overnight in 12-well plates at a density of 10^6 ^cells per well, serum starved for 5 h, and incubated with various concentrations of RIAA for 1 h. Cells were washed with PBS and lysed in lysis buffer containing 0.1% Triton X-100, 20 mM Tris (pH 8.0), 100 mM KCl, 1 mM DTT, 1 mM PMSF and protease inhibitor cocktail. Total cell lysates were electrophoresed, and incubated overnight at 4°C with primary antibodies of phospho β-catenin (Ser33/37), phospho-ERK1/2 (Thr202/Tyr204), phospho-p38 (Thr180/Tyr182) and phosphor-JNK (Thr183/Tyr185). These antibodies have been shown previously to be specific [11]. Secondary antibody linked to horseradish peroxidase (Amersham Biosciences) was incubated for 1 h at room temperature, after which proteins were visualized using the enhanced chemiluminescence (ECL) system from Pierce (Rockford, IL). For the loading control, the membranes were stripped and the blot analyzed by using anti-actin antibody.

### NF-κB driven luciferase activity

RAW 264.7 cells were sub-cultured in 96-well plates at a density of 7 × 10^4 ^cells per well and transiently transfected using SuperFect transfection reagent with an NF-κB or cAMP-responsive-element (CRE) firefly luciferase construct (SuperArray, Frederick, MD). After 2 days, cells were pre-incubated with various concentrations of RIAA or the NF-κB inhibitor parthenolide (10 μM) for 1 h in serum-free media, followed by 8 h LPS (1 μg/ml) stimulation. Luciferase activity was measured using Dual-Luciferase^® ^Reporter Assay System (Promega, Madison, WI) per the manufacturer's instructions. Transfection was normalized with constitutively expressing Renilla luciferase.

### Kinase assays

Kinase assays were performed at the Upstate Biotechnology (Dundee, UK). Briefly, GSK-3α/β activity was measured in the absence or presence of RIAA. In a final reaction volume of 25 μl the kinase of interest (5–10 mU) was incubated with 20 μM peptide substrate (YRRAAVPPSPSLSRHSSPHQS(p)EDEEE), 10 mM MgAcetate and [γ-^33P^-ATP] (specific activity approximately 500 cpm/mM) in the presence of 8 mM MOPS (pH 7.0) and 0.2 mM EDTA. The reaction was initiated by the addition of the 10 μM MgATP mix. After 40 min of incubation at room temperature, the reaction was stopped by the addition of 5 μl of a 3% phosphoric acid solution. 10 μl of the reaction was then spotted onto a P30 filtermat and washed 3 times for 5 min in 50 mM phosphoric acid and once in methanol prior to drying and scintillation counting. Detailed protocols are available online (at ).

### Nitrite/Nitrate

RAW 264.7 cells were sub-cultured overnight in 96-well plates at a density of 7 × 10^4 ^cells per well and incubated with various concentrations of RIAA for 1 h in serum-free media, followed by overnight LPS (1 μg/ml) stimulation. Nitrate/nitrite levels in the medium were measured using the Fluorometric Assay Kit (Cayman Chemical, Ann Arbor, MI) per the manufacturer's instructions.

### Osteoclastogenisis and tartrate-resistant acid phosphatase (TRAP) activity

RAW 264.7 cells were sub-cultured in 48-well plates at a density of 7 × 10^4 ^cells per well and incubated with various concentrations of RIAA in medium at a final concentration of 0.1% DMSO. Following overnight incubation, 50 ng/ml of soluble receptor activator of NF-κB-ligand (sRANKL) was added. After 2 days, medium and all reagents were replaced and incubation was continued for 3 more days. Cells were washed with ice cold PBS, lysed in 150 μl of 0.2% Triton X-100 in PBS, and TRAP activity was determined using a TRAP Kit from Sigma (Cat. #387A1). Briefly, 100 μl lysate was added to 100 μl of TRAP solution and incubated at 37°C for 1 h followed by measurement of absorbance at 555 nm. Protein concentration was estimated using BCA reagent (Bio-Rad) and final activity was normalized for equal protein.

### Chondrocytes and matrix metalloproteinase (MMP)-13 expression

The human chondrosarcoma cell line SW1353 was purchased from ATCC and maintained in L-15 medium in the presence of 10% FBS, according to manufacturer instructions. Cells were sub-cultured overnight in 96-well plates at a density of 8 × 10^4 ^cells per well. Following 1 h of incubation with various concentrations of RIAA, TNF-α or IL-1β (10 ng/ml) was added for 20–24 h and MMP-13 levels measured in medium using an ELISA kit (Amersham Biosciences), according to the manufacturer's instructions.

### Animal study

The study was performed at the Washington Biotechnology, Inc. (Simpsonville, MD) laboratories with approved standard protocol (CIA-MI). (i) Induction of collagen-induced arthritis: 6.5 ml of bovine type II collagen was emulsified with an equal volume of Complete Freund's Adjuvant (CFA, Chondrex, Redmond, WA, 4 mg/ml). Female DBA/1J mice (6–7 weeks) [2] were injected subcutaneously at the base of the tail with 50 μl of the emulsion (containing 100 μg type II collagen), with a booster injection after 21 days. After 7 days, mice that developed arthritis were used for the study. (ii) Experimental groups: Each group consists of 10 animals for (1) vehicle treatment – 50 μl distilled water/CFA emulsion, (2) celecoxib – 20 mg/kg, (3–5) RIAA – 250, 50 and 10 mg/kg, respectively, and (6) the "non-diseased" control. Test agents in 2% Tweeen-80 were administered daily by gavage (10 ml/kg). Treatment continued for 14 days and the arthritic index determined every other day. (iii) Determination of the arthritic index: Each paw was evaluated on the basis of a 4-point ordinal scale: 0 – no visible signs, 1 – edema/erythema of a single joint or digit; 2 – edema/erythema of 2 joints; 3 – edema/erythema of > 2 joints; 4 – severe arthritis of the entire paw and digits accompanied by ankylosis of the ankle and limb deformity. The index was calculated by summing from all four paws with a maximum score of 16. (iv) Histological evaluation: At day 42, mice were euthanized. One limb from each mouse was removed and preserved in 10% buffered formalin, decalcified, and subsequently trimmed so as to render a longitudinal section through the limb and digits. The specimens were processed, blocked, sectioned, stained with Haematoxylin and Eosin for microscopic examination. Soft tissue, bone and joint changes were evaluated using a standardized severity score whereby 0 = not present, 1 = minimal, 2 = mild, 3 = moderate and 4 = severe.

### Statistical analysis

SAS 9.0 (Cary, NC) was used for the statistical analyses. The in vitro data (NO inhibition, TRAP activity and MMP-13 inhibition) and the in vivo data (arthritis index and histological index) were analyzed using one-way ANOVA with Dunnett's post-hoc multiple comparisons in which the treatment groups were compared to the positive control; data are expressed as mean ± SEM. The significance level was at 0.05. No statistical tests were performed on data from a single experiment.

## Results

### Effects of RIAA on signaling pathways in LPS-stimulated RAW 264.7 macrophages

**Figure 1:**
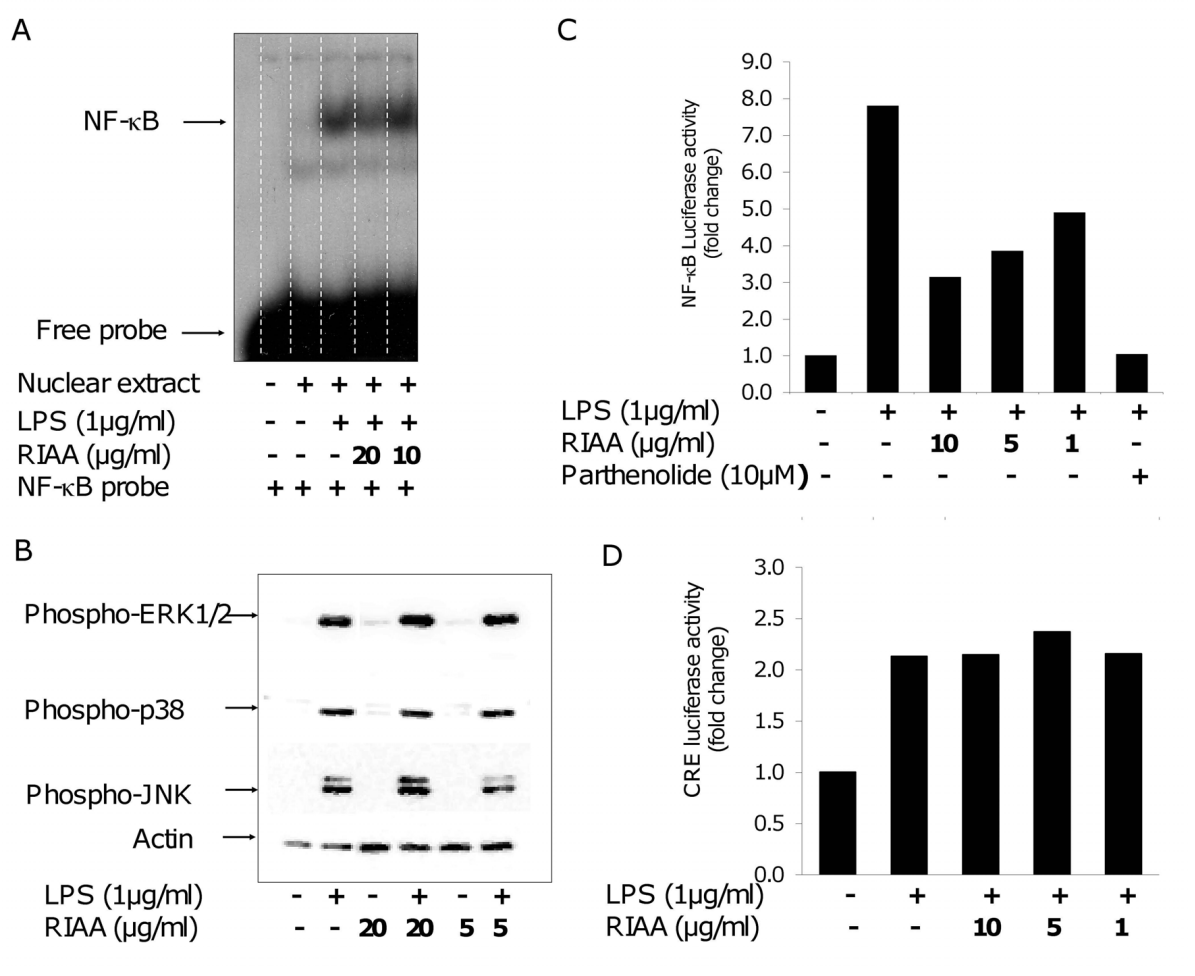
**Effect of RIAA on LPS-induced inflammatory signaling pathways in RAW 264.7 cells**. (A) Cells were incubated for 1 h with RIAA (0, 10 and 20 μg/ml) followed by LPS stimulation (1 μg/ml) for 2 h. Nuclear extract were analyzed for NF-κB binding by EMSA. (B) Cells were pre-incubated with RIAA (5 and 20 μg/ml) for 1 h and stimulated with LPS (1 μg/ml) for 1 h. Cell lysates were analyzed for phosphorylation of ERK1/2, p38 and JNK using western blot. (C) Cells transiently transfected with NF-κB firefly luciferase construct were incubated with RIAA (1, 5 and 10 μg/ml) or NF-κB inhibitor parthenolide (10 μM) for 1 h followed by LPS (1 μg/ml) stimulation for 8 h. The luciferase activities were determined and normalized with Renilla expression, and expressed as fold change compared to vehicle control. Data shown is representative of the experiment repeated several times. (D) Cells transiently transfected with CRE firefly luciferase construct were incubated with RIAA (1, 5 and 10 μg/ml) for 1 h followed by LPS (1 μg/ml) stimulation for 8 h. The luciferase activities were determined and corrected with Renilla expression, and expressed as fold change compared to vehicle control. Data shown is representative of the experiment repeated several times.

First, the effect of RIAA on NF-κB in vitro was investigated. RAW 264.7 cells were treated with LPS alone (1 μg/ml) or RIAA (10 and 20 μg/ml) followed by LPS incubation. Nuclear extracts were then analyzed by EMSA. RIAA at 20 μg/ml inhibited NF-κB to its consensus sequence as evidenced by reduced band intensity (Fig. 1A, lane 4). Next, the effect of RIAA on MAPK was investigated. Western blot analysis of total extract showed that, in the presence or absence of LPS stimulation, RIAA (5 and 20 μg/ml) had no effects on phosphorylation of ERK1/2, p38 and JNK in RAW 264.7 cells (Fig. 1B). Next, RAW cells were transfected with either NF-κB or CRE firefly luciferase construct, treated without or with RIAA (1, 5 and 10 μg/ml), and followed by LPS (1 μg/ml) stimulation. The luciferase reporter assays revealed that RIAA inhibited transactivation of NF-κB (Fig. 1C), but had no effects on that of CRE (Fig. 1D).

### Effects on the GSK-3 signaling pathway

**Figure 2:**
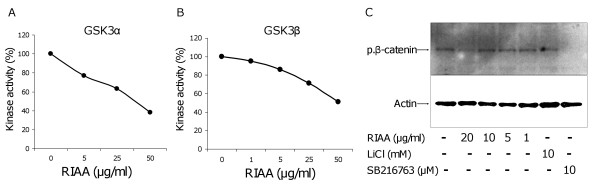
**Effect of RIAA on the inhibition of GSK3 pathway**. (A) GSK-3α was incubated with RIAA (0, 5, 25 and 50 μg/ml) and the kinase activity was determined. Data represent 1 representative experiment and are expressed as % activity. (B) GSK-3β was incubated with RIAA (0, 1, 5, 25 and 50 μg/ml) and the kinase activity was determined. Data represent 1 representative experiment and are expressed as % activity. (C) RAW 264.7 cells were incubated with RIAA (0, 1, 5, 10 and 20 μg/ml), or GSK-3 inhibitors LiCl (10 mM) and SB216763 (10 μM) for 1 h. Cell lysates were analyzed for the inhibition of phosphorylation of β-catenin by western blot.

GSK-3α/β kinase activities were evaluated in the absence or presence of RIAA (1 – 50 μg/ml) in cell-free kinase assays. We found that RIAA dose-dependently inhibited both kinases (Fig. 2A and 2B). We also measured phosphorylation of β-catenin, a known GSK-3 substrate, in RAW 264.7 cells by western blotting. Cells treated with RIAA for 1 h inhibited β-catenin phosphorylation (Fig. 2C).

### Effects on inflammatory markers associated with the NF-κB pathway

**Figure 3:**
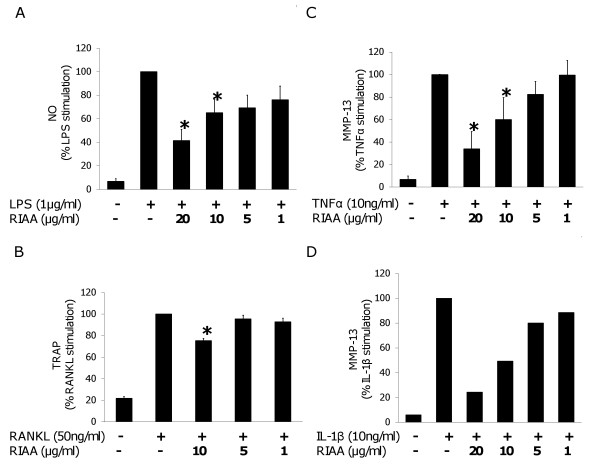
**Effect of RIAA on the inhibition of inflammatory markers**. (A) RAW 264.7 cells were incubated with vehicle or RIAA (1, 5, 10 and 20 μg/ml) for 1 h and subsequently stimulated with LPS (1 μg/ml) overnight. Nitrite/nitrate levels were measured in the medium and expressed as % activity over LPS stimulation. Data represent mean ± SEM (n = 6). (B) RAW cells were pretreated with vehicle or RIAA (1, 5 and 10 μg/ml) overnight and stimulated with RANKL (50 ng/ml) for transformation to osteoclasts for 5 days. TRAP activity was measured in the cell lysate and expressed as % activity over RANKL stimulation. Data represent mean ± SEM (n = 6). (C) SW1353 cells were incubated with vehicle or RIAA (1, 5, 10 and 20 μg/ml) for 1 h and stimulated with TNF-α (10 ng/ml) for 20–24 h. MMP-13 levels were measured in the medium and expressed as % activity over TNF-α stimulation. Data represent mean ± SEM (n = 5). *a significant difference (P < 0.05) in % activity compared to the respective positive control after Dunnett post-hoc adjustment. (D) Protocol same as (C) except IL-1β (10 ng/ml) was used instead of TNF-α. Data shown is that of a single experiment.

We investigated whether RIAA affected various inflammatory markers using established in vitro cell models [6,12,13]. First, in LPS-stimulated RAW 264.7 cells we found that RIAA dose-dependently inhibited NO levels, particularly at 10 and 20 μg/ml (Fig. 3A). Second, in RANKL-transformed osteoclasts/RAW cells, RIAA at 10 μg/ml inhibited TRAP activity (Fig. 3B). Third, in SW1353 cells, RIAA at 10 and 20 μg/ml inhibited TNF-α mediated MMP-13 secretion in a dose-dependent manner (Fig. 3C), and RIAA at 5, 10 and 20 μg/ml inhibited IL-1β mediated MMP-13 secretion (Fig. 3D).

### Inhibition on arthritis index in CIA model

**Figure 4:**
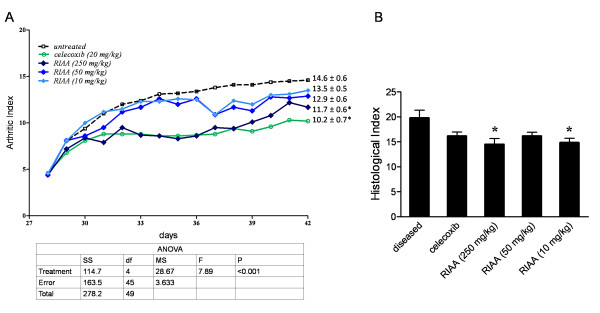
**Effect of RIAA in mice model of CIA**. The DBA/1J mice were injected intradermally with 100 μg of bovine type II collagen. After 21 days the mice were given a booster dose. CIA was fully developed at day 28 when treatments initiated and continued for the next 14 days. The arthritis index was assessed every other day. (A) The mean arthritis index for 3 RIAA treatment groups (10, 50 and 250 mg/kg body weight), celecoxib-treated group (20 mg/kg body weight) and the vehicle-treated control group. Day 42 data are expressed as mean ± SEM. Mice in the non-diseased group had an arthritis index of 0 throughout the experiment and therefore the data points were not displayed. (B) After evaluation of the arthritis index was completed at day 42, mice were anesthetized, and a histological examination was performed in 3 RIAA treatment groups, celecoxib-treated group and the vehicle-treated group (mean ± SEM). *a significant difference (P < 0.05) in histology score compared to diseased control after Dunnett post-hoc adjustment.

Mice with CIA were orally fed 10, 50, or 250 mg/kg-body-weight of RIAA, and symptoms of joint swelling were measured daily for 14 days and the arthritic index was calculated. We found that RIAA dose-dependently reduced the arthritis index, and at 250 mg/kg, their efficacy was similar to that of 20 mg/kg of celecoxib, the positive control (Fig. 4A). Joints from the animals were also evaluated histologically. RIAA at 250 mg/kg significantly reduced the histological index, but not celecoxib (Fig. 4B). Analyses of individual markers revealed that, compared to the diseased controls, animals receiving RIAA (250 mg/kg) showed significant reduction in joint destruction (1.90 ± 0.43 vs. 0.20 ± 0.20, P < 0.001), cartilage degradation (2.65 ± 0.26 vs. 1.85 ± 0.15, P = 0.003) and bone erosion (2.65 ± 0.15 vs. 1.75 ± 0.17, P = 0.002).

## Discussion

Inflammatory diseases such as RA are predominantly treated with NSAIDs or selective COX-2 inhibitors. Due to their well-known adverse effects in the gastrointestinal and cardiovascular systems when they are used long-term, researchers have been seeking novel candidates with safer modes of action. We found RIAA to be a promising anti-inflammatory candidate as it did not negatively impact gastrointestinal and cardiovascular biomarkers (e.g. fecal calprotectin and PGI-M/TXB_2 _ratio) commonly associated with NSAIDs [6]. To gain further understanding of RIAA's anti-inflammatory mechanism (s), we evaluated its effects on cell signaling pathways and in vitro inflammatory markers. We examined the effects of RIAA on NF-κB and MAPK in RAW 264.7 macrophages because these pathways activate transcriptional and post-transcriptional factors that upregulate pro-inflammatory genes, enzymes and cytokines [1,14,15]. We found that RIAA inhibited the LPS-activated nuclear NF-κB binding to its consensus sequence, which indicated a reduction in nuclear abundance of NF-κB (Fig. 1A). In addition, RIAA dose-dependently reduced the LPS-induced-transcriptional activity of NF-κB (Fig. 1C). On the other hand, RIAA did not affect the phosphorylation of ERK1/2, p38 and JNK in RAW cells (Fig. 1B), nor the transactivation of CRE (Fig. 1D), a known transcriptional factor regulated by MAPK in LPS-activated macrophages [16]. The data demonstrated that RIAA specifically inhibited NF-κB signaling pathway but not MAPK pathways.

In general, the activation of NF-κB is regulated at several levels, including IKK-mediated IκB degradation and nuclear translocation and DNA binding. During our screening we found that RIAA did not inhibit IKK (data now shown). Alternatively, post-translational modification of NF-κB-p65 regulates the function of NF-κB [3,17,18]; and through the p65 subunit, GSK-3 has been shown to regulate the production of pro-inflammatory cytokines. Therefore, we investigated the effects of RIAA on the GSK-3 signaling pathway. Both GSK-3α and GSK-3β were inhibited by RIAA (Fig. 2A and 2B), and phosphorylation of a known GSK-3 substrate, β-catenin, was inhibited by RIAA in RAW 264.7 cells (Fig. 2C). This mode of action is similar to a known GSK-3 inhibitor, SB216763, which also inhibited β-catenin [19]. It has been shown that β-catenin plays a significant anti-inflammatory role by reducing NF-κB activity [20]. Therefore, our data suggested that the anti-inflammatory mechanism of RIAA involves the inhibition of GSK-3/NF-κB pathway. Future assessment of the inhibition of β-catenin phosphorylation in vivo will further strengthen our in vitro finding.

We proceeded to investigate whether RIAA affected NF-κB-mediated pro-inflammatory mediators in various in vitro models, and found that it inhibited NO in LPS-stimulated RAW 264.7 cells; RANKL-mediated TRAP activity in transformed osteoclasts; and TNF-α/IL-1β-mediated MMP-13 expression in SW1353 human chondrosarcoma cells. It has been demonstrated that NO is involved in the pathogenesis of arthritis [21,22]; enhanced TRAP activity of osteoclasts increases bone resorption and contributes to bone loss [23]; and IL-1/TNF-α stimulate chondrocytes to increase production of MMPs and other degradative products [24-26]. These mediators cause bone erosion and cartilage degradation, and result in joint damage in RA [27]. Interventions targeting one or more of these mediators have been reported to reduce inflammation and degree of severity of RA, osteoarthritis and osteoporosis [23-25]; therefore, we tested the therapeutic effect of RIAA in mice with CIA. Two weeks of oral administration of RIAA at 250 mg/kg-body weight ameliorated joint damage in these mice as evidenced by significant reductions in the arthritis index and histology score. We found that the efficacy in reducing the arthritis index at this dose was similar to that of 20 mg/kg-body weight of celecoxib. Interestingly, however, celecoxib did not significantly reduce the histology score. This is likely due to the fact that celecoxib inhibits COX-2-mediated PGE_2 _synthesis evidenced by the reduced arthritis index, but does not inhibit NF-κB, an important mediator for bone and cartilage degradation.

Kinase inhibitors have shown beneficial effects in preclinical or clinical trials or in animal disease models for the treatment of cancer and autoimmune diseases [19,28,29]. For instance, GSK-3β inhibitors TDZD-8 and SB216763 reduced NF-κB mediated inflammation in rats with endotoxemia, and protected tissue damage in CIA mice. Our data demonstrated that RIAA inhibited GSK-3β/NF-κB mediated signaling pathways and inflammation, and reduced RA symptoms in CIA mice, suggesting that RIAA may have potential as an anti-inflammatory therapeutic.

## Competing interests

The study was funded by MetaProteomics, LLC, a subsidiary of Metagenics, Inc. that manufactures the commercial medical food for licensed healthcare professionals. All authors are employees of MetaProteomics.

## Authors' contributions

All authors participated in the concept and design of the study, and contribute to manuscript preparation. VRK and AD carried out the in vitro assays and performed the statistical analyses. GD carried out the animal study. All authors read and approved the final manuscript.
